# Unique roadside memorials: a lesson on road safety from Chile’s Atacama Desert Region

**DOI:** 10.5249/jivr.v16i1.1868

**Published:** 2024-01

**Authors:** David C. Schwebel

**Affiliations:** ^*a*^ Department of Psychology, University of Alabama at Birmingham, Birmingham, AL, USA.

## Commentary

Roadside memorials are displayed worldwide to pay homage to those lost in motor vehicle crashes, and scholars from a wide range of disciplines and geographic locations have discussed the cultural, religious, and psychological impact on passing motorists, victims’ families, and society.^1-8^ A small body of research examines the specific impact of roadside memorials on the safety of drivers passing by them; a recent systematic review^9^ identified four relevant studies, which together suggest roadside memorials have minimal impact on driver distraction or speed but may reduce red light violations among some drivers.^10-13^

Globally, most roadside memorials consist of either a simple marker, sometimes in the shape of a Christian cross, or a small shrine. These are the types of memorials studied in previous safety research.^9^ While traveling recently in the Atacama Desert region of Northeast Chile, I came across a different, less common, and more unique form of roadside memorial: destroyed vehicles left at the site of motor vehicle crashes. I witnessed burned trucks and shelled-out vehicles. In some cases, tires and other salvageable parts had been removed, but the carcassed remains of vehicles stood. Some were left upside-down. Others were burned, and many were crushed or dented. A few were visible from the roadside but lying below, down inclines or mountainsides; others were left standing directly on the roadway shoulder.

In all cases, experiencing a phenomenon similar to experimental findings from a videotaped experiment with small memorials in the form of white crosses,^10^ I found it difficult to resist inspecting and pondering the ruins, standing sadly as a memorial to those lost and reminding all passersby of the risks of driving quickly, distractedly, or intoxicated on the curvy and hilly roadways of the remote, mountainous region.

**Figure 1:**
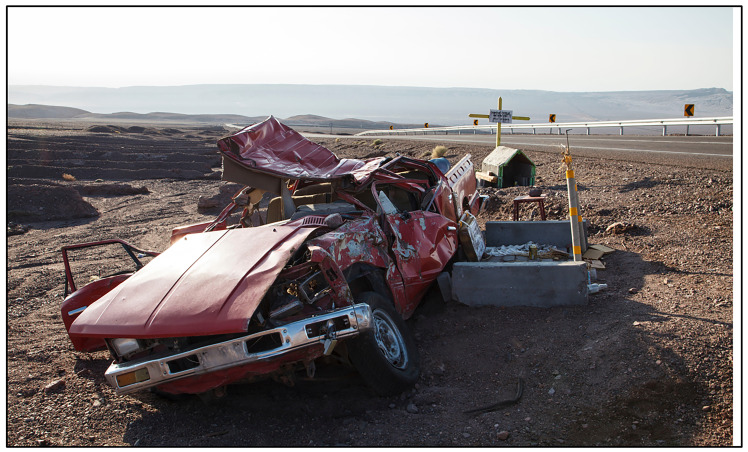
Photograph of wrecked vehicle and memorial along a highway in Chile’s Atacama Desert Region. Credit: Mauritius Images GmbH / Alamy Foto de stock.

As a traffic safety professional driving past the wrecked vehicles, I experienced a series of emotions. First, I was curious. How did that vehicle crash? Who was the driver? Were there passengers? And what became of them? In some cases, roadside memorials sadly communicated fatalities (See Figure 1). In other cases, it was unclear if some occupants had survived. After contemplating my curiosity, emotions of sadness emerged next. Many crashes were quite horrific-looking, and I remembered the anonymous victims, and feeling for their families.

Next came a key piece of behavior change for a traffic safety professional: I decelerated. My own vehicle deceleration triggered a transition from tourist to scientist. Might my own driving behavior replicate? Could this odd and unusual memorial to roadway victims – their wrecked vehicles – serve as a unique and special reminder of the risks of reckless driving? The abandonment of wrecked vehicles extends well beyond more traditional roadside memorials placed worldwide and studied in a small set of experiments,^9^ and I wondered whether the strategy might alter driver behavior and reduce traffic fatalities.

The World Health Organization estimates 1.3 million lives are lost annually in road traffic crashes,^14^ about 2,200 of them in Chile.^15^ Scholars widely recognize that preventive efforts will save lives, but the identification and implementation of cost-effective prevention strategies to reduce driver risk-taking and increase safety remains a vexing challenge to both policymakers and interventionists.

Behavioral science theory poses several effective strategies to alter health-related behavior like risky driving.^16^ Visually explicit roadside memorials incorporating abandoned and wrecked vehicles might prompt several of these strategies. First, they may lead to an increased perception of vulnerability; if drivers feel a risky behavior might lead to injury or death, they are more likely to change that behavior. Second, they illustrate the potential severity of a risky action. If drivers recognize a behavior like speeding might lead to serious injury or death, their motivation to change that behavior intensifies. And third, they reflect a form of peer influence. If someone else crashed – even an anonymous and unknown driver – it leads us to acknowledge it might happen to us as well and we therefore reduce risk-taking.

In short, the deliberate act of leaving ruined crashed vehicles at the site of their drivers’ demise sends a message for behavior change that is supported by health behavior theory and could yield more cautious driving. It increases vulnerability to risk, conveys the potential severity of that risk, and impacts perceived peer norms. Empirical tests of such initiatives are recommended, with any initiatives conducted carefully and sympathetically after gaining permission from the crash victim(s) or their surviving families as well as considering the impact on local wildlife.

If empirical tests prove effective and appropriate arrangements are made, other jurisdictions should take action to replicate the strategies in Chile’s Atacama Desert and reduce the toll of road safety injury and death on our global highways.

**Declarations: **This research was conducted without external funding. The author reports no conflicts of interest.
